# Effects of 6 weeks in-season flywheel squat resistance training on strength, vertical jump, change of direction and sprint performance in professional female soccer players

**DOI:** 10.5114/biolsport.2023.118022

**Published:** 2022-07-21

**Authors:** Javier Pecci, Alejandro Muñoz-López, Paul A. Jones, Borja Sañudo

**Affiliations:** 1 Department of Physical Education and Sport, University of Seville, Seville, Spain; 2 Departamento de Motricidad Humana y Rendimiento Deportivo, University of Seville, Seville, Spain; 3 School of Health Sciences, University of Salford. Salford, UK

**Keywords:** Soccer, Strength, Jump performance, Change of direction, Sprint ability, Flywheel

## Abstract

Flywheel resistance training devices (FRTD) is shown effective in improving strength, sprinting, jumping and changes of direction (COD) performance in male soccer players, however, this is not elucidated in female soccer players. We aimed to assess the effect of FRTD on these physical abilities in females soccer players. 24 professional female soccer players (age: 20.4 ± 2.6 years) were randomly assigned to a flywheel training group (FWTG) that trained twice a week for 6 weeks on a rotary inertia device with an initial volume of 3 sets of 6 repetitions and an inertia of 0,025 kg · m^-2^, increasing intensity and volume or a control group (CG) that did not performed any additional resistance training program. Concentric peak torque of the knee extensors (CONEXT) and flexors (CONFLEX), eccentric peak torque of the knee extensors (ECCEXT) and flexors (ECCFLEX) at 60° · s^-1^ on an isokinetic dynamometer, countermovement jump (CMJ) height, COD and 30-metres sprint were assessed. Significant time by group interactions were found in CONEXT (p = 0.002; η²p = 0.425), CONFLEX (p = 0.037; η²p = 0.22), ECCEXT (p = 0.002; η²p = 0.43) and ECCFLEX (p = 0.008; η²p = 0.334). No time by group effect was found in CMJ (p = 0.061; η²p = 0.182), COD (p = 0.067; η²p = 0.184) or sprint (p = 0.926; η²p = 4.979 · e^-4^). In conclusion, 6 weeks of flywheel squat training improved strength (especially eccentric strength) but not soccer-specific abilities such as jumping, changing of direction or sprinting in professional soccer players.

## INTRODUCTION

High-intensity actions, such as sprinting, jumping or changes of direction (COD) are decisive both in men’s and women’s soccer [1, 2]. These actions are becoming even more important in recent years, with the subsequent increment in the distance covered at high-intensities and the total number of sprints performed by soccer players [3, 4].

Women performance in high-intensity actions, such as sprinting or COD are differential factors in the competitive level, especially in high-speed running and sprinting due to its strong link to scoring chances [5]. These high-intensity actions (i.e. sprinting and COD), together with the countermovement jump (CMJ) were described as discriminatory variables of the competitive level in female soccer players, differentiating elite female soccer players from those of a lower level [5, 6]. Therefore, sprinting, COD and jump height are considered crucial skills in women’s soccer performance and in the game demands.

Lower-limb strengthening has been described as one of the main strategies to improve jump performance, COD and sprint time in soccer players [7, 8]. Moreover, and despite COD being a multifactorial ability [9], its performance is also related to strength development [10]. Consequently, due to the relevance of the strength levels in women’s soccer [11] and its aforementioned importance in sprint, COD and jump performance, improving these might be one of the main goals of a strength and conditioning (S&C) coach in women’s soccer.

In female athletes, physical performance was also significantly enhanced after resistance training [12], and especially in soccer [13]. These studies are usually based on traditional methods employing free weights (e.g., barbell) [14], weight stacks (e.g., pulley guided weight machine) [15] and/or the athletes’ body mass [16], which usually represent a constant external load that does not suit the athlete [17] (i.e., do not provide maximal resistance load during both movement phases). To overcome this issue, flywheel resistance training devices (FRTD) allows athletes to adapt the intensity to their effort due to the accommodated resistance from the flywheel [17], providing a nearly maximal resistance load during the concentric (CON) and eccentric (ECC) phase of the movement [17] due to their mechanical properties.

Interest in the use of FRTD has increased considerably [18]. FRTD consist of one or more disks connected to a shaft with a tether or a rope wrapped around [19]. Flywheel systems use kinetic energy generated during the CON phase of the movement to transfer it to ECC phase [17, 18]. Due to the greater force that ECC actions can produce with a reduced cost of energy [20] and the larger adaptations produced by resistance training based on ECC exercise such as in hypertrophy, strength and power [21], FRTD have been commonly used to develop specific and determinant abilities in team sport players, such as sprinting, COD or jumping [19, 22].

Longitudinal studies (i.e., 6–8 weeks resistance training programs) using FRTD have shown significant improvements in strength, COD, jump height or sprint performance in male soccer players [13, 22, 23]. Further, a recent systematic review [24] reported that a wide variety of training programs based on flywheel devices can improve strength, power, jump, and COD time in male soccer players of different competitive levels. However, it seems that 6–11 weeks are enough to significantly enhance these abilities.

Nevertheless, only three studies have analyzed the effects of resistance training with FRTD on female team sport players. One was focused on the effects of unilateral exercises on unilateral jump height, COD and sprint in volleyball and basketball players [25]. Another analyzed strength, speed, power and COD changes after flywheel squat resistance training on basketball players [26]. Only one has analyzed the effects of a training program based on FRTD in female soccer players [27], reporting significant improvements in the intensity of maximum accelerations and decelerations during soccer matches as well as the distance performed accelerating and decelerating at 3 m/s^2^. Therefore, previous studies performed different exercises and/or in different populations [25, 26] or analyzed very different variables from those assessed in the present study [27]. Despite the aforementioned importance of FRTD on strength and soccer-specific abilities in this population, to our knowledge, there are no studies assessing the effects of this training paradigm on these abilities in female soccer players following basic exercises such as squat. Evidence is needed for S&C coaches on the effects of training with FRTD in female soccer players to implement them in strength training programs. Consequently, the aim of this study was to analyze the effects of a 6 weeks in-season training program on lower-limb strength, sprint, COD and jump performance using a FRTD in professional female soccer players. It was hypothesised that 6 weeks of complementary flywheel squat resistance training would significantly improve strength levels, vertical jump height, COD and sprint time.

## MATERIALS AND METHODS

### Subjects

Twenty-four professional female soccer players (age: 20.4 ± 2.6 years; height: 1.62 ± 0.05 m; body mass: 58.7 ± 7.2 kg; body-mass index: 22.2 ± 2.4 kg · m^-2^; competition experience: 8.86 ± 3.36 years) belonging to the same club competing in the First National Division voluntarily participated in the study. During the season, the usual training volume was three training sessions per week on the soccer field of 90 to 120 minutes in addition to a competitive match throughout the weekend. Players were already familiar with traditional resistance training, but not FRTD. The study was conducted according to the Declaration of Helsinki, and the experimental protocol was approved by a local research ethics committee (CEI de los Hospitales Universitarios Virgen Macarena y Virgen del Rocío, Seville, Spain. Approval number: 2471-N-20). All players were required to sign an informed consent in which the entire evaluative and experimental process was detailed.

Players were excluded from the study prior to the initial evaluations if I) has suffered any joint or bone injury in the last six months, II) had programmed a change in her lifestyle during the evaluative or experimental phase of the study that could alter the results of the tests proposed for the evaluation or III) was pregnant.

### Design

The current study followed a randomized controlled trial model. Players were randomly assigned to a flywheel training group (FWTG; n = 12) or a control group (CG; n = 12). FWTG underwent a resistance training intervention in addition to the usual training on the soccer field, while CG continued their usual training sessions without performing flywheel resistance training or any complementary resistance training. Both groups performed the same training sessions on the soccer field. Players who did not complete at least 75% of the training program or who were unable to complete posttests (i.e. due to injury) were not included in the final analysis. Two FWTG players (i.e., one due to injury and another due to not having completed 75% of the training sessions) and two CG players (i.e., due to injury) were not included in the final analysis, resulting in a reduced sample size both in FWTG (FWTG; n = 10) and CG (CG; n = 10). Besides, one participant had very extreme scores on her COD data and was removed from the analyses as an outlier. The descriptive data of each group are shown in Table 1.

**TABLE 1:** Descriptive data of the players. Mean ± SD.

	FWTG (n = 12)	CG (n = 12)
Age (years)	20.8 ± 2.6	20.1 ± 2.6
Height (m)	1.61 ± 0.04	1.64 ± 5.6
Body mass (kg)	57.5 ± 7.3	59.9 ± 6.9
Body-mass index (kg · m^-2^)	22.15 ± 2.8	22.3 ± 2
Field position		
Goalkeeper (n)	1	2
Central back (n)	3	2
Full back (n)	2	2
Central midfielder (n)	3	2
Lateral midfielder (n)	2	2
Forward (n)	1	2

### Methodology

The testing protocol is shown in Table 2. Players were instructed to avoid vigorous exercise the day before testing. Field tests were carried out in the following order: CMJ, COD and sprint. Between tests, the players were allowed a 3-minute rest time. All players were previously familiarized with the evaluation protocol procedures due to their inexperience with the tests performed. During the execution of the tests, the players received strong verbal encouragement.

**TABLE 2:** Evaluation and experimental intervention schedule

	Week 1	Week 2–7	Week 8
Day 1	Body composition Isokinetic measurements Field tests familiarization	Experimental intervention	Isokinetic measurements

Day 2	Field tests: CMJ, COD and sprint	Experimental intervention	Field tests

*Isokinetic measurements.* A Biodex dynamometer (Biodex Medical System, Shirley, New York, USA) was used for the isokinetic measurements. The players were seated on the dynamometer with a hip angle of 85°. The lateral epicondyle of the femur was aligned with the axis of rotation of the dynamometer. A warm-up consisting of 3 sets of 10 body-weight squat repetitions was performed [23]. Concentric peak torque of the knee extensors (CONEXT) and flexors (CONFLEX) as well as eccentric peak torque of the knee extensors (ECCEXT) and flexors (ECCFLEX) were measured at 60° · s^-1^ [23].

Following two warm-up repetitions, each player executed 1 set of 5 repetitions at maximal intended effort, evaluating the extension and the flexion movements in pairs (concentric-concentric and eccentric-eccentric), with a 2-minute rest of passive recovery. Only the dominant leg, defined as the preferred leg to kick the ball [23] was evaluated.

*Field tests.* Before testing, the players conducted a warm-up consisting of 5 minutes of jogging at a self-selected pace, 2–3 minutes of lower-limbs mobilizations and 2 sets of 10 body-weight squats. Following, they conducted a specific warm-up: 1 set of 5 submaximal jumps (CMJ) and 2 submaximal efforts (COD and sprint). All the tests were carried out on the players’ usual training surface (artificial turf), except for the vertical jump due to the equipment used. The tests were performed with specific footwear for soccer practice.

*Countermovement jump test (CMJ).* Vertical jump height isolating the lower-limbs action was used as a representative measure of lower-limb impulsive strength [19]. Contact cells (Optogait, Microgate, Bolzano, Italy) were used to determine flight (t), with jump height determined from the formula: jump height = G*t^2^/8. Players were instructed to jump for maximum height with hands placed in their hips and without flexing the knees during the flight phase, as well as on landing. Each player performed three jumps, separated by a 2-minute passive recovery rest time. The best of the three trials was entered into the analysis. Results showed an *excellent* reliability in this test (Intraclass correlation coefficient -ICC- (3,1) = 0.951) from the three attempts performed.

*5+5 meters shuttle run-sprint test (COD test).* Players performed two maximum efforts, with 2 minutes of passive recovery between trials. One single beam timing gate (Microgate, Bolzano, Italy) was placed at the beginning to record the time to complete the test. The players had to sprint from the start, located 30 centimeters behind the timing gate to a line marked 5 meters apart and return to the starting position as quickly as possible, stepping beyond the line freely with the preferred foot of the player [9]. The best of the two trials was introduced in the final analysis. Results showed *excellent* reliability (ICC (3,1) = 0.911) from the two attempts performed.

*30-metres linear sprint test.* The evaluation of the linear sprint was carried out with the same photocells (Microgate, Bolzano, Italy), located both at the start and 30 meters away. The players had three attempts, separated by 3-minutes of passive recovery between them. The best of the three attempts was used for data analyses. Players were placed 30 centimeters behind the first photocell for the start of the sprint, with the preferred foot in a forward position and the toe of the foot on the line marked on the ground [23]. *Excellent* reliability was found for this test (ICC (3,1) = 0.945) from the three attempts performed.

### Experimental intervention

**FIG. 1:**
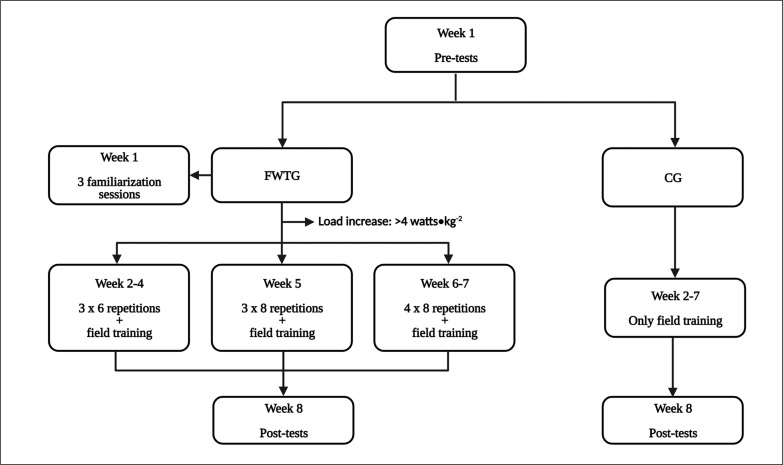
Resistance training program for the experimental intervention.

Figure 1 represents the intervention protocol. Prior to the intervention, three familiarization sessions were provided during week 1 to ensure squatting correct technique in parallel with the evaluation protocol. A FRTD with a horizontal cylinder-shaped shaft (Kbox 3, Exxentrix AB, Stockholm, Sweden) and a harness fixed to the shaft through a strap were used for the intervention. We used two different moments of inertia (inertia #1: 0,025 kg · m^-2^, #2: 0,050 kg · m^-2^) commonly used with female populations [28] that showed a significant loading using the same FRTD [29]. This allows to progressively increase the external load along the resistance training program, following the progression training principle [30]. Players were instructed to execute the CON phase as fast as possible, and consequently to decelerate the disk at the end of the ECC phase (e.g., thighs parallel to the ground). Players performed two resistance training sessions per week. The training sessions were separated by a minimum of 48 hours, coinciding with the usual on-field training days and prior to the main training soccer field session. At the beginning, players performed a 5-minute warm-up consisting of lower-limbs mobilizations and 1 submaximal set of 8 repetitions with inertia #1. During the experimental intervention, progression in training load was followed (see Figure 1). Players performed 2 submaximal repetitions prior to each set to speed up the disk. Rest time was 2 to 3 minutes between sets. During the training sessions, power was recorded using a rotary encoder (SmartCoach^TM^, SmartCoach Europe AB, Stockholm, Sweden). All players started with inertia #1, progressing to inertia #2 if they were able to generate a mean CON power > 4 watts · kg^-1^ in all repetitions of a set [13].

### Statistical analysis

Data is shown in the text as percentage of change (%) ± standard deviation (SD). First, we test the data normality using the Shapiro-Wilk normality test. Then, we compared the changes in Time (Pre vs Post) by Group (FWTG vs CG) using a two-way mixed ANOVA repeated measures test. In addition, we tested changes in Time and Group as main factors. If a significant interaction was observed, we performed an exploratory simple main effects analysis for Time factor, using Group as moderator (between-groups differences). We used the eta-partial squared (η²p) as a measure of the effect size. For statistical differences, we considered an alpha level of 0.05. We carried out all the analyses using the JASP software (JASP Team-2020, Version 0.14.1).

Our power analysis, based on the test performed (two-way mixed ANOVA repeated measures test), revealed a minimum of 16 participants in total to reach a statistical power of 80% with an alpha error of 0.05 and an effect size of 0.40 (corresponding to a large ƞ^2^_p_).

## RESULTS

**FIG. 2:**
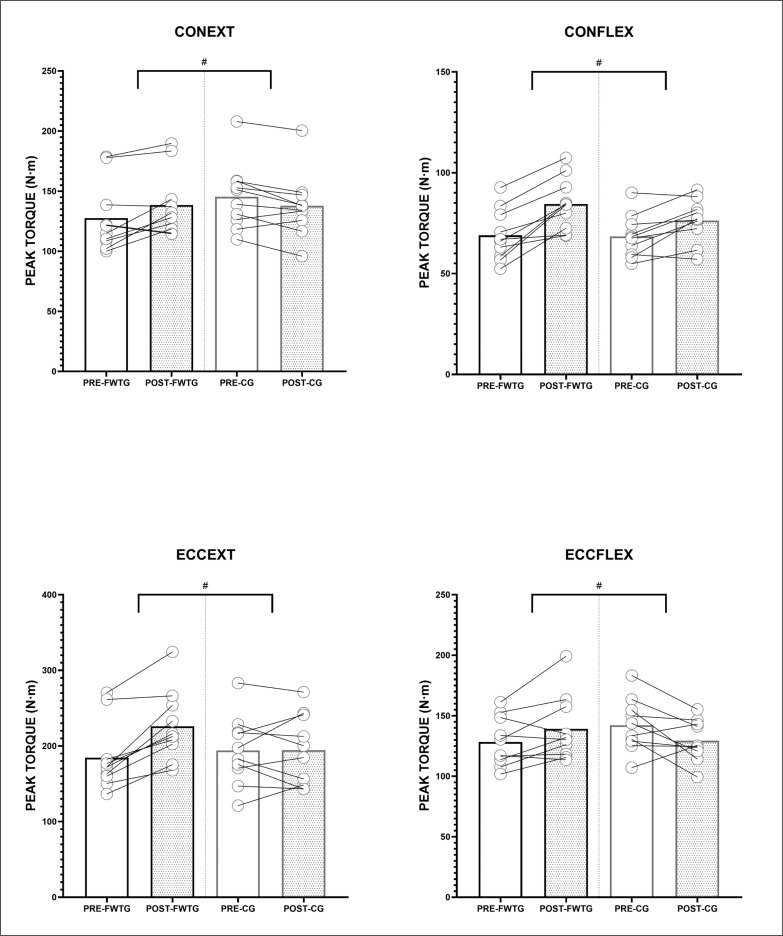
Pre-post changes in isokinetic measurements. CONEXT = Concentric peak torque of the knee extensors. CONFLEX = Concentric peak torque of the knee flexors. ECCEXT = Eccentric peak torque of the knee extensors. ECCFLEX = Eccentric peak torque of the knee flexors. FWTG = Flywheel training group. CG = Control group. # = Significant time by group interactions (p < 0.05).

FWTG showed an increase in CONEXT (+9.84% ± 12.32), CONFLEX (+23.91% ± 15.42), ECCEXT (+23.81% ± 13.71) and ECCFLEX (+8.51% ± 11.31) mean values. CG showed an increase in CONFLEX (+12% ± 11.27) and ECCEXT (+1.12% ± 14.77) mean values, with a reduction in CONEXT (-5.02% ± 6.77) and ECCFLEX (-7.78% ± 13.70) mean values. Time by group interactions were found in CONEXT (p = 0.002; η²p = 0.425), CONFLEX (p = 0.037; η²p = 0.22), ECCEXT (p = 0.002; η²p = 0.43) and ECCFLEX (p = 0.008; η²p = 0.334). Descriptive data of isokinetic measurements and individual changes are shown in figure 2.

The results of the field tests are shown on Table 3.

**TABLE 3:** Changes in field tests.

Test	Group	Pre (mean ± SD)	Post (mean ± SD)	Change (∆ %)	Changes in Time		Changes in Group		Changes in Time X Group	
					p	η^2^ p	p	η^2^ p	p	η^2^ p
CMJ (cm)	FWTG	26.08 ± 5.76	27.87 ± 7.78	5.61 ± 7.3	0.098	0.145	0.568	0.018	0.061	0.182
	CG	28.4 ± 3.02	28.28 ± 3.48	-0.38 ± 6.61						

COD (s)	FWTG	2.95 ± 0.18	2.91 ± 0.16	-1.46 ± 2.3	< 0.001	0.527	0.236	0.082	0.067	0.184
	CG	2.91 ± 0.13	2.79 ± 0.11	-3.98 ± 2.89						

Sprint (s)	FWTG	5.07 ± 0.32	5.04 ± 0.38	-0.55 ± 1.81	0.388	0.042	0.580	0.017	0.926	0.001
	CG	5 ± 0.26	4.96 ± 0.29	-0.59 ± 3.53						

CMJ = countermovement jump test. COD = change of direction test. FWTG = flywheel training group. CG = control group.

## DISCUSSION

Our results showed that 6 weeks of flywheel squat training significantly improved strength (CONEXT, CONFLEX, ECCEXT, ECCFLEX), but not CMJ, COD or sprint compared to traditional soccer field training.

Recently, Coratella et al. [23] evaluated the effects of 10 weeks of flywheel squat training in male soccer players and reported significant improvements in all isokinetic peak torque parameters in knee extensors and flexors. Further, Nuñez et al. [19] reported significant improvements in lower-limbs power in an all-out 8 repetitions test after 6 weeks of flywheel squat training in male soccer players using the same FRTD. Our results, although in female players, are in agreement with these findings, showing that 6 weeks of flywheel squat added to the usual on-field training are enough to induce significant and superior improvements in CONEXT, ECCEXT, CONFLEX and ECCFLEX. Therefore, we attribute the improvements and the differences reported in FWTG compared to CG to adaptations derived from the 6 weeks of flywheel squat training. It should be noted that greater effects were found in ECCEXT and ECCFLEX rather than CONEXT and CONFLEX, respectively. This finding could be attributable to the marked ECC component presented by FRTD, which showed greater muscle activation in the ECC phase of the movement than free weights [14].

Contrary to our hypothesis, our results have also shown that 6 weeks of flywheel squat training using a FRTD was not effective in significantly improving jump performance in female soccer players. Our results in CMJ changes are not in agreement with those reported by Coratella et al. [23], which reported a significant 8-week training effect in CMJ height, but not a significant inter-group change compared to the CG. Nonetheless, it is important to note that the CG performed a traditional back squat program as a part of the training protocol in the aforementioned study. In our study, CG did not perform any complementary resistance training, since our objective was not to compare this type of training (i.e., flywheel squat training) with other resistance training methodologies. Besides, these authors also showed a greater effect size in experimental group, suggesting that flywheel squat training programs produce superior adaptations in jump performance than traditional field soccer training alone. Similarly, De Hoyo et al. [22] showed substantial improvements in CMJ performance following a FRTD training program performing both half-squat and leg curl exercise in male soccer players. This study was carried out with a control group that did not perform any type of complementary resistance training, as in the present study. Nuñez et al. [19] also found that 6 weeks of flywheel squat was effective in improving jump performance. Thus, our results in CMJ performance in female soccer players are not in agreement with those previously reported in male soccer players [19, 22, 23]. This fact could be explained, as exposed by Pedersen et al. [32], due to the lack of high-intensity jumpings during field training sessions usually experienced in female soccer players. In this line, these authors argued that improving jumping or sprinting requires players to apply high levels of force at high velocities in addition to strength training. Men do get these stimuli with field training, but women do not, so it would be necessary to implement an additional jumping program to observe improvements in the CMJ in female soccer players.

Also contrary to our hypothesis, FWTG did not reach significant pre-post improvements in COD or sprint. Despite having improved ECC strength to a greater extent and its importance in deceleration and COD abilities [33], our results did not show a superior effect in the players that performed the flywheel squat exercise. Jones et al. [33] demonstrated that greater ECC strength levels are associated with a better 180º COD performance in high level female soccer players, due to being able to handle the greater loads experienced with faster approach velocities. These authors also highlighted the importance of developing several abilities such as strength, speed and technique for improving COD, so this fact could explain the lack of improvements in COD performance in the present study. The lack of improvements in COD, based on the *theory of specificity*, can also be attributed to non-specific stimulus of the flywheel squat in the sagittal plane and the horizontal vector. De Hoyo et al. [31], in line with our results, found no significant improvements in COD in the group that performed the squat exercise in the frontal plane. Nuñez et al. [19] also showed unclear changes in COD 180 degrees following a 6 weeks flywheel squat training program, reporting greater improvements in COD through unilateral exercises better than bilateral exercises (e.g., squat). Hence, unilateral exercises in FRTD could lead to greater improvements in COD ability rather than solely bilateral exercises as in the present study. Thus, due to the importance of determinants to improve the COD (i.e., strength, speed, COD technique and performing unilateral exercises), we suggest that these factors could explain the lack of improvements in COD after the flywheel squat training program in the present study, but more research is necessary. Therefore, S&C coaches as well as future research should take into account the specificity as well as the characteristics of the exercise for the periodization of training and thus be able to determine if these factors could result in a substantial increase in COD performance.

We also hypothesized that 6 weeks of flywheel squat could improve 30-metres sprint time. Vertical force production is directly associated with sprint performance, especially with the ability to sprint over longer distances (> 20 metres) [34]. Thus, performing vertically-directed exercises such as squat could result in significant improvements in sprint performance [34, 35]. In addition, higher levels of lower-limbs strength are strongly associated with better sprint performance [7, 36, 37]. Nonetheless, despite having improved strength levels on knee extensors and flexors, FWTG did not significantly improve 30-metres sprint time. The lack of improvements could be explained through the *theory of specificity* due to the non-specific stimulus of the flywheel squat in the sagittal plane and the horizontal vector. In this line, Loturco et al. [35] highlighted that vertically-directed exercises would be more associated with top-speed phases, which often reached in > 40-metres sprints. Our results in 30-metres sprint, although in females, are in agreement with those reported by Coratella et al. [23], which did not show significant pre-post changes after 8 weeks of flywheel squat training in male soccer players.

The only study carried out with female soccer players [27] analyzed very different variables from those evaluated in the present study. It showed significant improvements in accelerations and decelerations, thus, it is possible that FWTG had a better performance in these actions at the end of the training program, especially in the last minutes of the game as reported in the aforementioned study. Nonetheless, possible improvements in the acceleration ability does not translate into improvements in 30-meter sprint and in the COD at maximal velocity as shown by the results of the present study.

However, more studies with female soccer players are needed to corroborate the effects of training using FRTD. Moreover, it is necessary to assess training programs based on FRTD in a more holistic sprint and COD development programme (i.e. including sprint/ COD technique training, plyometrics, etc.).

Several practical applications should be highlighted from this study: 1) Strength (especially ECC strength) can significantly improve using FRTD in professional female soccer players, 2) 6 weeks of flywheel squat was not enough to improve soccer-specific abilities such as jumping, COD or sprinting and 3) to improve CMJ, COD or sprint other stimulus are necessary.

It should be noted that this paper has some limitations. The first limitation is the criteria to increase inertia (i.e., load). This criteria was used in male soccer players [13], consequently it is necessary to evaluate and establish specific criteria for selection of initial training load and load increase in females. The present training program progression was mainly focused on volume increase, whereas inertia only progressed if the player was able to overcome the aforementioned threshold. Different training programs with load increase based on inertia increments could potentially have greater strength adaptations [12]. Hence, the lack of improvement in high-velocity movements as those evaluated in the present study. Finally, the higher training volume in FWTG as a consequence of supplemental resistance training added to the usual on-field training means that the two compared groups have not been subjected to the same training volume. However, the purpose of the present study is not to compare the effects induced by resistance training based on flywheel devices with respect to other devices used in strength training.

In contrast, this study has several strengths that should be high-lighted. The first strength is the evaluation of high-intensity and soccer-specific actions such as vertical jump, change of direction and sprint after a resistance training program based on a FRTD for the first time. Another strength is sample homogeneity, all belonging to the same club and undergoing the same field training. The results of the present study could be also considered a strength, since it provides important information for S&C coaches such as flywheel squat could improve strength levels but not soccer-specific abilities, so the implementation of flywheel squat in the resistance training program for female soccer players should consider these limitations.

## CONCLUSIONS

Results of the present study show that 6 weeks of in-season biweekly flywheel squat training significantly improved CON and ECC strength of the knee extensors and flexors in professional female soccer players, highlighting the improvement in ECC strength, but not jump height, COD or sprint time. These results suggest that resistance training performing flywheel squat improve professional female soccer players strength, with interesting adaptations in ECC strength, but not soccer-specific abilities. Thus, coaches could benefit from the improvements that training programs based on FRTD produce, leading to superior adaptations than traditional soccer field training alone in strength levels.
